# Assortative Mating between European Corn Borer Pheromone Races: Beyond Assortative Meeting

**DOI:** 10.1371/journal.pone.0000555

**Published:** 2007-06-20

**Authors:** Laurent Pélozuelo, Serge Meusnier, Philippe Audiot, Denis Bourguet, Sergine Ponsard

**Affiliations:** 1 Laboratory of Applied Entomology Graduate School of Agricultural and Life Sciences, The University of Tokyo, Tokyo, Japan; 2 Centre de Biologie et de Gestion des Populations (CBGP), UMR 1062, Institut National de la Recherche Agronomique (INRA), Campus International de Baillarguet, Montferrier-sur-Lez, France; 3 Laboratoire Dynamique de la Biodiversité, UMR CNRS 5172, Université Paul Sabatier, Toulouse, France; University of Exeter, Cornwall Campus, United Kingdom

## Abstract

**Background:**

Sex pheromone communication systems may be a major force driving moth speciation by causing behavioral reproductive isolation *via* assortative meeting of conspecific individuals. The ‘E’ and ‘Z’ pheromone races of the European corn borer (ECB) are a textbook example in this respect. ‘Z’ females produce and ‘Z’ males preferentially respond to a ‘Z’ pheromone blend, while the ‘E’ race communicates *via* an ‘E’ blend. Both races do not freely hybridize in nature and their populations are genetically differentiated. A straightforward explanation would be that their reproductive isolation is a mere consequence of “assortative meeting” resulting from their different pheromones specifically attracting males towards same-race females at long range. However, previous laboratory experiments and those performed here show that even when moths are paired in a small box – i.e., when the meeting between sexual partners is forced – inter-race couples still have a lower mating success than intra-race ones. Hence, either the difference in attractivity of E *vs.* Z pheromones for males of either race still holds at short distance or the reproductive isolation between E and Z moths may not only be favoured by assortative meeting, but must also result from an additional mechanism ensuring significant assortative mating at close range. Here, we test whether this close-range mechanism is linked to the E/Z female sex pheromone communication system.

**Methodology/Principal Findings:**

Using crosses and backcrosses of E and Z strains, we found no difference in mating success between full-sisters emitting different sex pheromones. Conversely, the mating success of females with identical pheromone types but different coefficients of relatedness to the two parental strains was significantly different, and was higher when their genetic background was closer to that of their male partner's pheromone race.

**Conclusions/Significance:**

We conclude that the close-range mechanism ensuring assortative mating between the E and Z ECB pheromone races is unrelated to the difference in female sex pheromone. Although the nature of this mechanism remains elusive, our results show that it is expressed in females, acts at close range, segregates independently of the autosome carrying *Pher* and of both sex chromosomes, and is widely distributed since it occurs both in France and in the USA.

## Introduction

Moth sex pheromones are volatile chemicals emitted by members of one sex (usually females), to which members of the other sex respond by directed flight, courtship and possibly mating. Almost four hundred sex pheromone components have been documented so far for moth species [1], and they can be combined into an even greater diversity of blends (http://phero.net/). Individual components can be shared by several species – especially closely related ones (*e.g.*, [2]–[4]). Nevertheless, the exact combination and relative amounts of these components seem to characterize most species uniquely [5], [6]. Members of the responding sex are usually narrowly tuned, with respect to both sensory equipment and behavioral response, to the precise blend released by conspecific emitters (*e.g.* [7]). It has thus been argued that sex pheromone communication systems can serve Lepidoptera systematics in a similar way to male and female genitalia morphology: because they are directly involved in specific recognition between mating partners, they can still delineate taxa even when these are so closely related that they are hardly distinguishable by any other phenotypic character [8].

The evolution of differences in pheromone communication systems is thought to facilitate and possibly even cause moth speciation [5], [6], [9]–[14] by promoting “assortative meeting” – the uneven probability of encounters between different types of individuals [15] – of conspecific moths. Indeed, the production and recognition of specific sex pheromones promote reproductive isolation between taxa [16]–[18] by causing them to be spatially segregated in the wild. Closely related taxa with pheromone polymorphism and partial reproductive isolation may be in the process of speciating *via* such a mechanism. The ‘E’ and ‘Z’ pheromone races of the European corn borer (ECB), *Ostrinia nubilalis* Hübner (Lepidoptera: Crambidae), are a textbook example of such taxa [18], [19]. Indeed, these two races use two different blends of the E and Z isomers of the long-chain, unsaturated Δ11-tetradecenyl acetate (Δ11-14:OAc). ‘E’ females release and ‘E’ males preferentially respond to an ‘E’ blend composed of Z11-14:OAc and E11-14:OAc in a 1∶99 to 4∶96 ratio [20], [21], while ‘Z’ females and ‘Z’ males use a ‘Z’ blend with a 99∶1 to 97∶3 ratio [20], [21]. The female offspring of hybrid crosses emit an intermediate 35∶65 Z∶E blend, to which hybrid males seem to be preferentially attracted [22], [23]. The differences in blend emitted by females, in electrophysiological response of the male antennae, and in male behavioral response are determined by at least three loci: *Pher* [22], [24], [25], *Olf* [23], [26] and *Resp* [25], [27], respectively. *Resp* is sex-linked, while both other loci are autosomal but segregate independently from each other [22], [23], [25].

The E and Z races co-occur in several locations of the species' range: the USA [21], [28], [29], France [30]–[32] and Italy [33]. Field studies showed that the proportion of hybrids between E and Z moths is often much lower than what could be expected when considering the frequency of the two races at local or regional scales (USA: [34]–[36]; France: [31], [37]). As laboratory experiments show no disadvantage in the offspring of hybrid crosses, this low frequency of hybrids is likely to result from a high level of assortative mating [38]. Accordingly, allozyme or DNA allele frequencies reveal a weak but significant overall genetic differentiation between both races in the USA [36], [38]–[40] and in France [30], [32], [37], [41], [42]. An appealing explanation for such a situation would be that both races are currently diverging genetically – and may eventually form two distinct species – as a result of reproductive isolation caused by the assortative meeting induced by their different Z/E11-14:OAc-based sex pheromone communication systems [38].

The level of spatial segregation between E and Z-race adults in the wild is still unclear. Indeed, wind-tunnel experiments showed that ECB males are more likely to take off and fly towards a same-race source of female sex pheromone than to a source of the opposite blend. In such experiments, pheromone sources are typically located at a few meters' distance [27]. Also, pairs of pheromone traps baited with the E and the Z pheromone blend preferentially attracted E- and Z-race males, respectively, when placed at <50 m distance [32]. More generally, it has been shown that males of various moth species are able to discriminate between sources of their own species' pheromone and other odours (eg, incomplete or off-ratio pheromone blends: [43], [44]) or pheromone antagonists (eg, [45], [46]) even when these sources are placed at a few centimeters' distance. All this suggests that, if E and Z females are spatially segregated when they emit their pheromones, E- and Z-race males should be able to distribute themselves accordingly. On the other hand, adult males and females caught with nets over several tens of meters in aggregation sites – i.e. in places where the ECB is known to mate preferentially and where males and females gather in high densities [47] – sometimes proved to be mixtures of E- and Z-race moths [37], suggesting that at least certain aggregation sites may be used simultaneously by the two races and that there is a sufficient degree of spatial overlap to offer large opportunity for hybridization between moths of the E and Z races. Still, results from laboratory experiments – including those presented here – suggest that such hybridization events remain rare. Indeed, even when moths are paired in the same box during several days, inter-race crosses have a lower mating success than intra-race crosses (USA: [48]; France [49, this study]). Hence, it would seem that at least two mutually non-exclusive mechanisms may contribute to reproductive isolation between the E and Z races of the ECB: an assortative meeting ensured by their difference in female sex pheromones and an additional mechanism acting at close range, which is yet to be discovered.

One possibility is that the E- or Z11-14:OAc blends are not only implicated in long-range attraction of ECB males of either pheromone race, but also causes them to mate preferentially with same-race females at short-range. Alternatively, the short-range assortative mating might be due to a set of pheromones other than the E or Z11-14:OAc blends, or even to a completely different mechanism that does not involve any kind of pheromonal communication system. Further, the genetic basis of this unknown mechanism may be either linked or unlinked to *Pher*, *Olf* and *Resp*, the three (unlinked) loci involved in the specific production and recognition of the sex pheromones.

The aim of this paper was to test whether, at short-range, the mating success of females with males of both races is linked to the sex pheromone they emit. In a preliminary experiment, we examined whether we could increase the mating success of inter-race couples by placing them into boxes where we attempted to mimic the atmosphere of a mixed aggregation site by additionally placing either two virgin females or a rubber septum loaded with synthetic ECB sex pheromone into the box, releasing the Z/E11-14:OAc blend of the male's race. Such approach has been successfully used in *Heliothis* [50]. However, as reported below (Results – Experiment 1), both treatments failed to cause any significant increase in mating success. In a second experiment (Experiment 2), using E and Z strains originating either from France or from the USA, we found that the level of short-range mating success of females with E and Z males is not linked to the sex pheromone blend they emit. Indeed, when placed individually with 1 male into a small plastic container during 3 nights, full-sisters emitting different sex pheromones (obtained by appropriate crosses and backcrosses) did not show any difference in mating success with males of a given race, while females with identical pheromone types but otherwise different genetic backgrounds displayed substantial differences in mating success with males of either race.

## Results

### Experiment 1

The purpose of this preliminary experiment was to see whether the lower mating success of E and Z males paired with a female of the opposite race could be restored by the addition of female sex pheromone of their own race. The mating success of intra- and inter-race crosses was evaluated on individuals from French Z and E strains, in the absence *vs.* presence of an additional source of sex pheromone. These sources were either two additional virgin females, or a lure releasing a typical E or Z blend of synthetic Δ11-14:OAc. In the absence of any extra source of sex pheromone, inter-strain pairs had – as expected – a much lower mating success than intra-strain pairs (2.4% and 8.6% *vs.* 51.3% and 64.4% respectively, Table 1). However, the presence of two virgin females or that of lures releasing the sex pheromone blend of the male's strain both failed to increase the mating success in inter-strain pairs (Table 1). The supplementary source of pheromone even significantly *reduced* the mating success in two cases: the E×E and the Z×Z crosses in the presence of E and Z lures, respectively (Table 1).

**Table 1:** Mating success in different types of crosses between the E and Z strains from France in the presence – in a separate compartment communicating *via* a grid – *vs.* in the absence of either a rubber septum loaded with synthetic pheromone or two virgin females belonging to the same strain as the male.

Cross Type^a^	Additional Source of Pheromone	*n*	Percent Females Mated^b^
E×E	none	45	64.4^a^
	E lure	45	13.3^b^
	Z lure	51	43.1^a^
Z×Z	none	80	51.3^a^
	E lure	45	31.1^a^
	Z lure	45	8.9^b^
	2 Z females	11	72.7^a^
E×Z	none	93	8.6^a^
	Z lure	43	9.3^a^
	2 Z females	45	13.3^a^
Z×E	none	85	2.4^a^
	E lure	45	2.2^a^
	Z lure	10	0.0^a^
	2 E females	44	0.0^a^

a Cross types are described as female×male.

b Within each cross type, only percentages followed by a different letter were significantly different from each other (Fisher's exact test, *p*<0.05).

### Experiment 2

**Figure 1:**
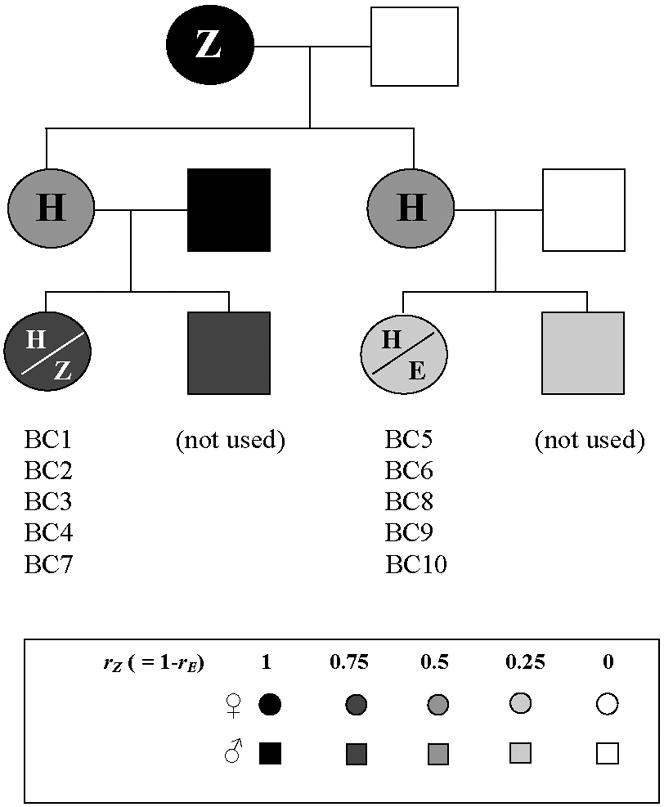
Experimental design for crosses and backcrosses performed with the E and Z strains to obtain the different lines – except BC11 – used in Experiment 2 and reported in Table 2. The BC11 line was obtained by crossing a female from the E strain with an F1 male from a female E×male Z cross. *r*_E_ and *r*_Z_ are the coefficient of relatedness of one female with E and Z parental strain, respectively.

By means of crosses and backcrosses (Figure 1) between the E and the Z strains we established in our laboratory, we obtained groups of females emitting similar pheromone blends – *i.e.*, Z, E or H (hybrid) type pheromones – but differing in their respective levels of average genetic similarity with the parental strains. For instance, H-type F1 females obtained from E×Z crosses had a coefficient of relatedness (*r*) of 0.5 with both parental strains (E and Z), whereas H-type backcross females obtained from F1×E backcrosses were more (*r* = 0.75) related to the E strain and less (*r* = 0.25) related to the Z strain – or *vice versa* if they were obtained from F1×Z backcrosses. Conversely, all backcross females within a particular line, being full-sisters, were equally related to both parental strains but consisted of two groups emitting different pheromones – H and E for F1×E or E×F1 backcrosses, and H and Z for F1×Z backcrosses.

**Table 2:** Backcrosses performed with the E and Z strains from France and from the USA.

Origin of the Parental Strains	Backcross			Pheromone Type of Full-Sisters (*n*)				*p*-value^d^
	Female	Male	# Line	E	H	Z	Undetermined^c^	
France	F1^a^	Z	BC1	-	56	52	28	0.770
			BC2	-	47	45	33	0.917
			BC3	-	5	4	0	1.000
			BC4	-	5	23	0	<10^−3^
	F1^a^	E	BC5	19	13	-	3	0.377
			BC6	20	9	-	1	0.061
USA	F1^a^	Z	BC7	-	8	4	19	0.194
	F1^a^	E	BC8	14	10	-	0	0.541
			BC9	92	43	-	18	<10^−4^
			BC10	79	44	-	35	0.001
	E	F1^b^	BC11	21	24	-	0	0.766

a Obtained from female Z×male E crosses.

b Obtained from a female E×male Z cross.

c Due either to a poor extraction of the pheromone compounds or to an insufficient quality of the chromatograms.

d *p*-values of two-tailed binomial tests comparing the observed proportions of E:H and Z:H pheromone types within each line with the 50∶50 expected under the assumption that pheromone types are determined by *Pher*, an autosomal locus with two codominant alleles: *Pher^E^* and *Phe^z^* [22], [24].

We generated a total of 11 lines – 6 using the French E and Z strains and 5 using the US E and Z strains – with 1 to 4 lines per backcross type (Table 2). As expected, gland washes of females from F1×E and E×F1 backcrosses contained either the E or the H pheromone type, whereas those from F1×Z contained either the Z or the H pheromone type. Most – 8 of 11 – backcrosses did not show any significant departure from the expected 50∶50 proportion of either E-type∶H-type or Z-type∶H-type females among the backcross offspring (Table 2). The fact that no female obtained from a backcross to the E strain (or, respectively, to the Z strain) was ever found to emit a Z-type (or, respectively, an E-type) pheromone is an indirect confirmation that the E and Z parental strains used in this study were fixed for the *Pher^E^* and the *Pher^Z^* allele, respectively, at *Pher*, the autosomal locus determining the E, H or Z type of the sex pheromone emitted by females [22], [24]. The fact that, in addition, the 3 remaining backcrosses (BC4, BC9 and BC10, Table 2) all included both H- and either Z- or E-emitting females indicates that their deviation from 50∶50 is unlikely to be due to one of the parents not having the expected genotype at the *Pher* locus, since this would have produced either 100% E-, Z- or H-emitting females, or a 25∶50∶25 blend of E-, H- and Z-emitting females respectively. The most likely explanation for these deviations is thus small sample size (BC4) or that pheromone determination failed more often for H- than for E- or Z-emitting females (BC9 and BC10), which is unlikely to have biased our results (see below).

If the Z/E 11-14:OAc ratio in the pheromone emitted by females determined their mating success with males of a given race, the expectation would be that, within each line, Z-emitting (or, respectively, E-emitting) backcross females should have a higher mating success with parental Z-strain males (or, respectively parental E-strain males) than their H-emitting full-sisters. However, we detected no significant difference between groups of full-sisters emitting different pheromone blends, be it in tests conducted among French or among US strains (Table 3a, multiple Fisher's test: χ^2^ = 4.85, *df* = 6, *p* = 0.563 and χ^2^ = 4.23, *df* = 6, *p* = 0.645 for the French and the US strains, respectively). This result also holds when tests conducted with French and US E-strain males are analyzed separately from tests conducted with French and US Z-strain males (multiple Fisher's test: χ^2^ = 3.21, *df* = 6, *p* = 0.782, and χ^2^ = 5.88, *df* = 6, *p* = 0.437 for E- and Z-strain males, respectively).

**Table 3:** Mating success comparisons between (**a**) full sisters – hence displaying the same coefficients of relatedness to the parental strains – but producing different pheromone types and (**b**) females emitting similar pheromone types but with different coefficients of relatedness to the two parental strains.

Origin of the Parental Strains	Mating Pairs					Expected Mating Success^b^	Percent Mating Success		*p*-value^d^	Model Validation^e^	Overall *p*-value^f^ (χ^2^; *df*)^f^
	A Females		B Females		Males^a^		A Females % (*n*)^c^	B Females % (*n*) c			
	Pheromone Type	Line(s)	Pheromone Type	Line(s)							
(**a**) Different Pheromone Types but identical Coefficients of Relatedness											
France	H	BC1	Z	BC1	Z	A = B	78.4 (51)	84.3 (51)	0.612	**Yes**	0.563 (4.85; 6)
	H	BC2, 3 & 4	Z	BC2, 3 & 4	E	A = B	40.4 (52)	29.0 (69)	0.245	**Yes**	
	H	BC5 & 6	E	BC5 & 6	Z	A = B	50.0 (22)	60.5 (38)	0.589	**Yes**	
USA	H	BC7	Z	BC7	E	A = B	62.5 (8)*	75.0 (4)*	1.000	**Yes**	0.645 (4.23; 6)
	H	BC8, 9 & 11	E	BC8, 9 & 11	Z	A = B	42.9 (77)	54.0 (124)	0.147	**Yes**	
	H	BC10	E	BC10	E	A = B	76.7 (43)	78.9 (76)	0.820	**Yes**	
(**b**) Identical Pheromone Types but different Coefficients of Relatedness											
France	Z	Z strain	Z	BC1	Z	A>B	84.0 (50)	84.3 (51)	1.000	No	0.015 (27.83; 14)
	Z	Z strain	Z	BC2, 3 & 4	E	A<B	12.0 (50)	29.0 (69)	0.042	**Yes**	
	E	E strain	E	BC5 & 6	Z	A<B	36.0 (50)	60.5 (38)	0.031	**Yes**	
	H	BC1	H	F1^g^	Z	A>B	78.4 (51)	68.0 (50)	0.267	Yes	
	H	BC5 & 6	H	BC1	Z	A<B	50.0 (22)	78.4 (51)	0.025	**Yes**	
	H	BC5 & 6	H	F1^g^	Z	A<B	50.0 (22)	68.0 (50)	0.189	Yes	
	H	BC2, 3 & 4	H	F1^g^	E	A<B	40.4 (52)	48.0 (50)	0.550	Yes	
USA	Z	Z strain	Z	BC7	E	A<B	54.0 (50)	75.0 (4)*	0.620	Yes	<0.001 (38.63; 14)
	E	E strain	E	BC10	E	A>B	92.3 (52)	78.9 (76)	0.049	**Yes**	
	E	E strain	E	BC8, 9 & 11	Z	A<B	28.0 (50)	54.0 (124)	0.002	**Yes**	
	H	BC10	H	F1^g^	E	A>B	74.4 (43)	86.3 (51)	0.190	No	
	H	BC7	H	F1^g^	E	A<B	62.5 (8)*	86.3 (51)	0.125	Yes	
	H	BC10	H	BC7	E	A>B	76.7 (43)	62.5 (8)*	0.404	Yes	
	H	BC8, 9 & 11	H	F1^g^	Z	A<B	42.3 (77)	68.0 (50)	0.007	**Yes**	

a E and Z males used in the mating pairs were always taken from the E and Z strains, respectively.

b Under the assumption that the difference in mating success between strains is controlled by at least one locus independent from *Pher* (see text for further explanations).

c Percentage of mated females. *n*: number of tested pairs. *: low number of replicates (*n*<10).

d *p*-values of a two-tailed Fisher's exact test of the null hypothesis that A females' mating success = B females' mating success. Individuals from different lines were pooled as indicated.

e **Yes**: the result fits the expectation listed in column b with Fisher exact test statistical validation. Yes: the result fits the expectation but is not validated statistically. No: result against expectation.

f Fisher's test for multiple comparisons for tests conducted on the lines of the same geographic origin. Fisher's test for multiple comparisons conducted either with males of the E-race or with males of the Z-race (regardless of geographic origin) yielded χ^2^ = 3.21, *df* = 6, *p* = 0.782 for E-race males, and χ^2^ = 5.87, *df* = 6, *p* = 0.438 for Z-race males in Table 3a and χ^2^ = 23.82, *df* = 14, *p* = 0.048 for E-race males, and χ^2^ = 42.65, *df* = 14, *p*<0.0001 for Z-race males in Table 3b.

g Obtained from female Z×male E crosses.

The alternative hypothesis is that the mating success of females is not determined *per se* by the type of pheromone they emit – at least in the close-range settings used here –, but rather by their average genetic similarity – *i.e.*, by their coefficient of relatedness (*r*) – with the strain of the male they are paired with. If so, E-emitting females resulting from an E×E intra-strain cross would be expected to show a higher (or, respectively, a lower) mating success with E-strain males (or, respectively, Z-strain males) than E-type females resulting from F1×E or E×F1 backcrosses. Similarly, ‘pure’ Z-strain females would be expected to show a higher mating success with Z-strain males and a lower mating success with E-strain males than Z-type females resulting from F1×Z or Z×F1 backcrosses. Indeed, consistently with these predictions, the experiments conducted with French E and Z strains (Table 3b, multiple Fisher's test: χ^2^ = 27.8, *df* = 14, *p* = 0.015) and those conducted with US E and Z strains (Table 3b, multiple Fisher's test: χ^2^ = 38.63, *df* = 14, *p*<0.001) both revealed significant differences. This result also holds when tests conducted with French and US E-strain males are analyzed separately from tests conducted with French and US Z-strain males (multiple Fisher's test: χ^2^ = 23.8, *df* = 14, *p* = 0.048, and χ^2^ = 42.7, *df* = 14, *p*<0.0001 for E- and Z-strain males, respectively).

Testing the significance of these results using multiple Fisher's tests – as we did – maximizes statistical power, but it implies that these results are considered to be independent tests of a single, common hypothesis. Alternatively, one could also decide to individually test each of the predictions listed in Table 3. This would imply considering that some of these predictions could conceivably be true while others would – simultaneously – be false. For instance, genetic similarity might influence mating success when females are paired say with French Z- but not when they are paired with US Z-strain males. Thus, we also separately tested 20 different implications of the assumption that the ability of a female to mate with an E- or a Z-race male has a genetic determinism segregating independently of *Pher*: twelve tests supported our predictions (*p*<0.05), six tended to support them (but with a *p*>0.05 level of significance), and two yielded non-significant (*p*>0.05) trends opposite to our predictions (Table 3). There was no obvious tendency for the tests to yield more non-significant results when conducted on strains from a particular geographic origin (USA or France), or with males of a particular race (Z or E).

Failures in pheromone type identification could have biased our conclusions, but we consider this unlikely. Pheromone analyses were performed after females had been tested for their propensity to mate. As mating typically causes lepidopteran females to temporarily stop or reduce their sex pheromone emission [51], we probably failed to identify the pheromone type more often for mated females than for virgin ones. Unsurprisingly, there were more mated females among those for which pheromone identification failed than among those for which it succeeded (Table S1; Fisher's multiple test over all lines, χ^2^ = 55.26, *df* = 14, *p*<10^−4^). This could potentially have biased the comparisons reported in Table 3a. Indeed, if one of the groups had had a higher propensity to mate, it would presumably have ‘lost’ the most numerous (mated) females to the ‘undetermined pheromone type’ category, and hence ended up with the most strongly underestimated mating success, thereby possibly preventing us from detecting an existing difference. Nevertheless, such bias is unlikely for two reasons.

Firstly, among the three lines where the proportion of H *vs.* E or Z pheromone emitting females significantly departed from 50∶50, one (BC4) included no female with undetermined pheromone type (Table 2), and two (BC9 and BC10) would still yield the same conclusion if we conservatively added all ‘undetermined’ mated females to the group with the pheromone type closest to that of the male partners' race (*i.e.*, to the group expected to show the highest success if mating were influenced by pheromone type) and all ‘undetermined’ non-mated females to the category with the most different pheromone type. Indeed, for BC9, 16 additional mated females would increase the mating success of category A to 51.6%, and 2 unmated ones would decrease that of category B to 53.1%, which would still not be sufficient for the former to be higher than the latter. For BC10, the mating success of category A and B would become 74.9% and 85.4%, respectively, but the difference would remain non-significant (Fisher's exact test: *p* = 0.159). The *p*-value of the overall test for US strains in Table 3a would become 0.273, which remains above the 0.05 threshold of statistical significance. The same applies to BC5 and BC6 (four mated females would increase the mating success of category A to 57.7%, which is still less than 60.5%). Among the three remaining lines (BC1, BC2 and BC7), such conservative calculation would affect the conclusion, but as the proportion of Z *vs.* H females among those determined was not significantly different from 50∶50 for those lines (Table 2), there is no reason to believe that the ‘undetermined’ category contained a higher proportion of H-type females than the ‘determined’ category. Secondly, the significant differences reported in Table 3b are unlikely to be an artifact due to non-exhaustive pheromone determination, as there is no reason to believe that they would have been systematically those expected under our alternative hypothesis simply by chance.

One or several locus (or loci) segregating independently of *Pher* [22], [24], thus seem(s) to be driving the mating success of females with males of the two pheromone races. For parsimony, we will further discuss our results as if there was only one locus – henceforth referred to as *Am* for ‘assortative mating’ – although our results provide no evidence with respect to the number of loci involved.

As there is no crossing-over in female Lepidoptera [52], [53], this *Am* locus must be located on a different chromosome than *Pher*. Also, *Am* is not located on the Z or on the W sex chromosome. Indeed, if the genetic determinism of mating success were completely Z-chromosome-linked, Z- and E-emitting females obtained from F1×Z or F1×E backcrosses would have the same mating success as females of the Z and E parental strains, respectively, because their Z-chromosome is inherited from their pure-strain father. Such prediction can be tested by comparing the mating success of pure-race males with F1 and backcross females on one hand (except BC11 females, which result from an E×F1 cross and may therefore have either ‘E-type’ or ‘Z-type’ Z chromosomes), and pure-strain females carrying the same respective type of Z chromosome on the other hand (when available: indeed, the mating success of French E-strain males with E-strain females and of US Z-strain males with Z-strain females have not been estimated in our study and are thus unavailable for comparisons). However, F1 and backcross females showed a significantly lower (or, respectively, significantly higher) mating success than the pure-strain females of the same (or, respectively, the opposite) pheromone type as that of their male partner's strain (Table S2; multiple Fisher's tests: χ^2^ = 28.69, *df* = 6, *p*<0.001 and χ^2^ = 29.57, *df* = 6, *p*<0.001 for French and US individuals, respectively).

Finally, it is unlikely that *Am* is located on the W chromosome. First, this sex chromosome generally carries very little functional genes [54], [55]. Second, if the genetic determinism of mating success were entirely W-chromosome-linked, all F1 and all backcross females except BC11 would have the same mating success as females of the Z parental strain. Indeed, all F1 females result from Z×E crosses and therefore carried a ‘Z-type’ W chromosome, which they transmitted to their backcross daughters (BC1 to BC10). For the same reasons, BC11 backcross females carry an ‘E-type’ W chromosome and would be expected to show the same mating success as pure E-strain females. However, F1 and backcross females showed a significantly lower (or, respectively, significantly higher) mating success than that of pure-race females of the same (or, respectively, the opposite) pheromone race as the males used for the test (Table S2; multiple Fisher's tests: χ^2^ = 18.54, *df* = 4, *p*<0.001 and χ^2^ = 27.63, *df* = 4, *p*<0.001 for French and US individuals, respectively).

Finally, one could imagine that, while *Pher* is the major locus determining the Z-, E- or H-type of the pheromone, *Am* is a ‘secondary’ locus marginally modifying the proportion of E and Z isomers in the blend. Löfstedt et al. [23] suggested the possible existence of such ‘modifier’ genes. Zhu et al. [56] detected small but heritable differences in the exact percentage of E isomer in the blend emitted by certain females. They attributed part of this polymorphism to the existence of more than one *Pher^Z^* allele and another part to a minor modifier locus, the exact nature of which remained elusive. The former polymorphism was detected because hybrid F1 females had an E:Z isomeric ratio close to either 65∶35 or 80∶20, and the hybrids of certain backcross lines showed a bimodal distribution of their E:Z isomeric ratio. However, none of our 11 backcross lines showed any significant departure from unimodality in the isomeric ratio of the blend emitted by H-type females (Hartigan tests [57], *p*>0.1 for all lines). Therefore, there is no evidence for the presence of more than one *Pher^Z^* and one *Pher^E^* allele in the strains we used. Similarly, only one among six backcross lines with sufficient sample sizes (*n*>12) to be tested showed a significant difference between the percentage of E isomer in the blend emitted by successfully mated *vs.* non-mated H-type females (Student's *t*-test, BC2: *p* = 0.025, BC1, BC5, BC9, BC10 and BC11: *p*>0.3). This difference was small (*ca*. 3%) and would not remain significant after a Bonferroni correction for 6 comparisons (*p* = 0.141). Thus, small differences – if any – in pheromone blend that might be caused by minor loci marginally influencing the exact E:Z isomeric ratio in ECB female sex pheromones are unlikely to account for the differences in mating rate we observed.

## Discussion

### Assortative meeting and assortative mating

Pheromones facilitate meeting between potential mates. Indeed, the E or Z pheromone blends attract ECB males at long range, as suggested by wind tunnel experiments [27] and shown by the successful use of such blends to bait agricultural monitoring traps [58], [59]. This may contribute to the reproductive isolation of the E and Z moths by causing a spatial segregation of the two races. Indeed, ECB males are selectively attracted to a source of the pheromone blend of their own race in the field, even when sources of the two blends are placed at a distance ≤50 m from each other [32]. Therefore, even where both races are sympatric at regional scale, fine-scale differences in the spatial distribution of adult females releasing their pheromones might result in a corresponding micro-allopatry of adult males, thereby making same-race crosses more likely than hybrid ones. Although – as far as we know – such micro-allopatry has never been formally documented, it is likely to occur, at least in France where the two races feed on different host plants [30], [31], [41], [42], unlike in the USA [28] and Italy [33] where both feed on maize. Pheromone differences may thus contribute to assortative mating between the E and Z race by promoting an assortative meeting.

From these observations and similar ones on taxa displaying pheromonal polymorphism (*e.g.*, [6], [10]–[12], [60]), it is tempting to infer that assortative meeting promoted by pheromone differences accounts for reproductive isolation between groups using different pheromones. Nevertheless, in the ECB, even in the absence of assortative meeting – i.e. when moths are paired in small cages during several days – inter-race crosses have a lower mating success than intra-race crosses (see Table 3 and [48]). This contrasts, for instance, with results obtained for *Zeiraphera diniana* (Lepidoptera: Torticidae) and *Trichoplusia ni* (Lepidoptera: Noctuidae), two other models for studying pheromone polymorphism and genetic divergence, where no reproductive barrier was detected at close range between different pheromone races [12], [14]. This suggests that the reproductive isolation between E and Z moths is not only ensured by assortative meeting but also, at close range, by at least one additional short-range factor contributing to assortative mating.

Our results show that this factor is independent from the difference in E/Z11-14:OAc female sex pheromone blends. First in inter-race crosses performed in Experiment 1, we were unable to restore the propensity to mate by adding a source of female sex pheromone blend corresponding to the male's pheromone race (Table 1). Most importantly in Experiment 2, we found no difference in the mating success of groups of full-sisters emitting different pheromones while significant differences were observed between groups of females emitting the same pheromone but with otherwise different genetic backgrounds (Figure 1, Table 2 and 3). We therefore hypothesize that a heritable factor different from *Pher* – the autosomal locus that determines the E/Z11-14:OAc ratio emitted by females [22] – contributes to assortative mating between the E and Z ECB pheromone races. Although the nature of this factor remains elusive, our results further show that it is expressed in females, acts at close range, segregates independently of the autosome carrying *Pher* and of both sex chromosomes (Table S2), and is widely distributed, since it occurs both in France and in the USA. This factor might have a polygenic basis (see ‘Results’ section) but, for parsimony, we discuss our results as if there were only one, and call this hypothetical locus *Am*.

### Is *Am* another locus of the Δ11-14:OAc-based communication system?

The E and the Z races are known to share a large amount of polymorphism at allozyme [38], [39], [41], [42] and DNA [40] level, but also to differ at two diagnostic loci in addition to *Pher*: *Olf* [22], [26] and *Resp* [22], [25], [27]. *Am* could be suspected of being one of them. It cannot be *Resp*, as *Resp* is sex-linked whereas *Am* is not (Table S2). The other candidate, *Olf*, is known to control the organization of the olfactory sensillae in ECB male antennae [23], [26]. However, as our results suggest that *Am* is expressed in females whereas ECB female antennae have very little such sensillae – [61] as cited in [62] – one would further have to assume that *Olf* has a different function in females. *Am* may or may not be the same locus as *Olf* – appropriate crossing experiments could tell – but there is no particular indication that its function in females is related to the Δ11-14:OAc-based communication system. Finally, *Am* could be suspected of being a modifier that marginally alters the E/Z11-14:OAc ratio mainly determined by *Pher* in the Δ11-14:OAc blend emitted by females [23], [56], but again we failed to detect any indication in our data that this might be the case (see ‘Results’).

### Has *Am* anything to do with a pheromone-based communication system?

In the present study, we were interested in the influence of the E/Z11-14:OAc ratio emitted by females on their mating success with males of the E or Z pheromonal race. However, *Am* could also cause females to emit another pheromonal component, *a priori* unrelated to Δ11-14:OAc. For instance, the Z11-16:OAc might be a candidate because it is frequently found in the pheromone blend released by E race females in both Italy and France, but rarely in that released by Z race females [31], [33]. However, it is not synergist to E/Z11-14:OAc at long distance [33] and no evidence has been found so far that this component elicits any behavior in males, so that its status as a *pheromone* component is questionable. A more detailed chemical analysis and component identification of the odorant blend released by F1 and backcross females might help identifying other candidate pheromone components, but we noticed no obviously variable peaks in the vicinity of the two Δ11-14:OAc isomers in the chromatograms we examined. Similarly, *Am* might encode a close-contact, non-volatile pheromone – *e.g.* a cuticular hydrocarbon [63] – but as the male courtship behavior does not involve any physical contact before mating, this, again, seems dubious.

In sum, while such possibility cannot be completely dismissed, there is no reason to believe that *Am* determines the emission of a pheromonal component by females more than any other component of a mate-recognition mechanism (*e.g.*, female response to a male sex pheromone [64], [65], to a male acoustic signal [66] or to some other trait correlated with male quality [67], or female emission of a signal yet to discover).

Finally, rather than in a mate-recognition signal, *Am* might be involved in fine behavioral differences common to both sexes and promoting assortative mating, such as synchronicity in the circadian sexual activity [48]. Detailed comparisons of the behavioral sequence leading (or not) to mating among intra- and inter-races couples and couples involving hybrid individuals are needed to identify candidate functions of *Am* and direct further investigations on gene expression.

### Conclusion

In sum, our results suggest that while sex pheromone differences might be a driving force in assortative mating *via* assortative meeting, one or several other mechanism(s) may also significantly contribute to assortative mating between closely related species. In the case of *Ostrinia nubilalis* pheromone races, differences in the Z/E11-14:OAc-based female sex pheromone communication system may have facilitated, and possibly even initiated divergence by promoting assortative meeting between the two ECB pheromone races. However, they may just as well have arisen at a later stage, once races were already differentiated due to factors ensuring assortative mating even at close range. When DNA sequences of the corresponding genome regions become available, comparing the sequence divergence of *Am* and of *Pher, Resp* and *Olf* may provide interesting insights into this question.

## Material and Methods

### French strains

The E strain was established from approximately 50 diapausing larvae collected from mugwort (*Artemisia vulgaris* L.) near Paris (Ile de France, France, 48°46′N, 02°04′E) and Lille (Nord-Pas de Calais, France, 50°63′N 3°07′E) in spring 2004. From previous results on populations collected across France, individuals collected on mugwort are known to use the E pheromone blend [30]–[32].

The Z strain was founded with approximately 100 pupae taken from an outbred strain reared at INRA-Le Magneraud (Surgères, Poitou-Charentes, France, 46°10′N, −0°75′E). This strain originated from wild larvae collected from corn (*Zea mays* L.) in the vicinity of Surgères. Individuals from this strain use the Z pheromone type, as shown by previous female gland analyses and male wind tunnel experiments [31].

### US strains

The E and Z strains were the UZ and BE strains established by Roelofs and colleagues at the New York State Agricultural Experimental Station (NYSAES) in Geneva, NY, from larvae and pupae collected from corn fields at Bouckville (NY, 42°89′N, −75°55′W) in April 1994 and at Geneva (NY 42°87′N, −76°98′W) in May 1996. The BE and UZ strains have been maintained ever since and are known for using the E and Z pheromone types, respectively [25], [40], [56]. We established these two strains in winter 2003–2004 in our lab from more than 300 eggmasses of each strain, which were kindly provided by C. Linn Jr.

### Rearing and crosses

All strains were reared at 22±2°C under a L:D 16:8h photoperiod. Larvae were fed on a standard artificial corn-based diet [68] and male and female pupae were kept separately, so that adults were all virgin prior to being used either to perform the crosses and backcrosses summarized in Figure 1 or to evaluate mating success as described below. We aimed at obtaining at least one line for each type of cross and backcross. For each line, a male and a female of the corresponding strains were placed in a cylindrical plastic box (8.5 cm diameter, 11 cm deep) containing a wad of moist cotton and a 15×1.5 cm strip of paper for resting and for oviposition. We collected as many eggmasses as possible, until the female died. Larvae obtained from these eggmasses were reared in the standard conditions described above.

### Mating success

For both experiments and all types of crosses, virgin females and males emerged since <24 h were paired and allowed to mate during 3 consecutive nights at 22±2°C and under a L:D 16:8h phototoperiod. In Experiment 1, either 2 additional 2-3d-old virgin females, or one commercial lure (Biosystem, Herblay, France) were placed into the box in which mating took place. The virgin females were placed into a small plastic container pierced with holes allowing the release of their sex pheromone but preventing any mating. Commercial lures were rubber septa loaded each with 100 µg of either the Z or the E pheromone blend. These Z and E pheromone blends contained the typical ratio of 97∶3 and 3∶97 Z and E11-14:OAc, respectively. After each experiment all reusable devices were bathed during one night and cleaned up with a medical detergent (Franck Lab SA, St Quentin en Yvelines, France) to remove any traces of sex pheromone.

At the end of the 3^rd^ night, females were killed and dissected to determine their mating status – virgin or mated – according to the content of the *bursa copulatrix* of their genital duct. Indeed, the sperm and nutritious substances transferred by males during mating form an easily recognizable solidified structure, the spermatophore (usually one per mating event [69]) that is later used by the female to fertilize her oocytes. Thus, the mating success for a given category is the percentage of females that were found to carry at least one spermatophore.

### Pheromone analyses

We analyzed the sex pheromones of the female offspring of the 11 backcrosses used in Experiment 2 and described in Table 2. Before dissecting the female to determine her mating status (see above), its pheromone gland was extruded by gentle pressure on the abdomen, excised and immediately immersed for 20 min in 20 µl of 99% grade hexane (Prolabo, Fontenay sous Bois, France) for pheromone extraction. Samples were stored at −20°C until aliquots of 3 µl were analyzed with an HP 5870 Series II gas chromatograph (Hewlett Packard, Palo Alto, CA, USA) equipped with a flame ionisation detector, a split/splitless injector, an automatic injector (Agilent 6890) and an apolar HT-5 (Supelco, Bellefonte, PA, USA) capillary column (25 m×0.32 mm Internal Diameter (ID), 0.1 µm fiber thickness). The carrier gas was helium at a speed of 1 ml.min^−1^. Injector and detector temperatures were 225°C and 295°C, respectively. The initial oven temperature was 120°C (we later found out that this temperature was probably too high for splitless injection with hexane as the solvent: a temperature 15°C below the boiling point of the solvent, i.e., 69−15 = 54°C for hexane, in the present case, might have yielded a better sensitivity (C. Löfstedt, *pers. com.*)). After 0.45 min, this temperature was increased at a rate of 15°C min^−1^ until it reached 190°C, then at a rate of 20°C min^−1^ until it reached 280°C. Solutions of the pure synthetic E or Z11-14:OAc isomers (Biosystem, Herblay, France) were used as standards to determine the retention time of these two main components of the ECB sex pheromone blend. Each chromatogram was read blindly (*i.e.*, without information on the parents or mating status of the corresponding female) and independently by three of us (PA, DB and SP). When chromatograms were found to be of insufficient quality, up to three additional analyses were performed for each extract. Due either to a poor extraction of the pheromone compounds or to an insufficient quality of all three chromatograms, the pheromone type of some females remained undetermined. For the other females, at least one chromatogram per female allowed unequivocal determination of the pheromone type. Females were assigned to the Z, E or Hybrid (H) type when the ratio between the height of the Z and the E11-14:OAc peaks was comprised between 95∶5 and 100∶0, 0∶100 and 5∶95 and 15∶85 and 50∶50, respectively.

Löfstedt et al. [23] and Zhu et al. [56] found indications for the existence of a ‘modifier’ locus that changes the exact proportion of the E isomer in the E:Z11-14:OAc pheromone blend emitted by females, which is mainly determined by *Pher*. We tried to detect the possible existence of such small differences by testing the percentage of E11-14:OAc isomer in the blend emitted by H-type backcross females with Hartigan tests [57] for unimodality, as described below. We also examined whether such small differences were likely to have affected the propensity of H-type females to mate by comparing the average proportion of the E isomer in the blend emitted by H-type females that had mated successfully with that of same-line H-type females that had failed to do so with Student's *t*-tests, as described below.

### Statistical analysis

When the pheromone type could not be determined, the female was excluded from the mating success analysis. We checked whether the proportion of H-emitting *vs.* E- or Z-emitting females in any given backcross significantly departed from that expected for a character determined by an autosomal locus with two alleles [22] by comparing the observed proportion with a theoretical proportion of 50∶50 by means of a binomial test. In Experiment 2, differences in mating success were first tested using two-tailed Fisher's exact tests. Due to a low number of offspring per line, backcross females producing the same pheromone type and paired with the same type of male (E or Z) were pooled. We further tested for differences in mating success by using a two-tailed Fisher's exact test for multiple comparisons. In this case, we calculated an observed χ^2^ value equal to −2⋅Σ*_i_*ln*p_i_*, where *p_i_* is the Fisher's exact test's probability obtained for the *i*-th paired comparison, and compared it with a theoretical value in a χ^2^ table with *df* = 2⋅*i* degrees of freedom [70], [71]. Such multiple analyses were applied for an overall comparison of the mating success of (1) females (full sisters) displaying a similar coefficient of relatedness (*r*) to the parental strains but emitting different pheromone types and (2) females with similar pheromone types but with different coefficients of relatedness (*r*) to the parental strains. In addition, it was applied separately to results obtained for French and for US strains and for Z and E strains.

To test for the possible presence of an ‘E-enhancer’ allele in our strains, we conducted Hartigan's [57] dip-test for unimodality on the percentage of the E11-14:OAc isomer in the blend emitted by all backcross H-type females within each line (6 tests, as BC3, BC4, BC6, BC7 and BC8 had too few hybrid F1 females – *n*≤10 – for the test to be meaningful). We also conducted one- and two-tailed Student's *t*-tests to see whether the percentage of the E11-14:OAc isomer in the blend emitted by H-type females was higher for those that had successfully mated with an E-male than among those that had not, or lower for those that had successfully mated with a Z-male than among those that had not. These tests were conducted separately for the six lines with *n*>10. We used one-tailed tests when the differences were as expected – *i.e*. when the percentage of the E11-14:OAc isomer was higher (or, respectively, lower) in mated *vs.* non-mated H-type females when paired with E (or, respectively, Z) males.

## Supporting Information

Table S1 Mating success of females for which the pheromone type could or could not be characterised in the different backcross lines. The backcrosses indicated in the first column are those described in Table 2. There were no undetermined females in BC3, 4, 8 and 11.
(0.04 MB DOC)

Table S2 Comparison of the mating success of pure-strain (A) females with that of F1 and backcross females that have a father (B) or a mother (C) of the same strain. A and B (respectively C) females are expected to have an identical mating success if Am is Z-chromosome (respectively W-chromosome) linked. The backcrosses are those described in Table 2.
(0.06 MB DOC)
