# Supplementary material for: Assortative Mating between European Corn Borer Pheromone Races: Beyond Assortative Meeting
Source: PLoS One. 2007 Jun 20;2(6):e555. doi: 10.1371/journal.pone.0000555 (PMC1891084; doi:10.1371/journal.pone.0000555)
Supplement: Table S1 — Mating success of females for which the pheromone type could or could not be characterised in the different backcross lines. The backcrosses indicated in the first column are those described in Table 2. There were no undetermined females in BC3, 4, 8 and 11. (0.04 MB DOC) [file pone.0000555.s001.doc]

|  | Pheromone Type | | | | |  | *p*-value^b^ | Overall  *p*-*value*^c^  (*χ²*; *df*) |
| --- | --- | --- | --- | --- | --- | --- | --- | --- |
| Backcross Line | Determined^a^ | |  | Undetermined | |  |  |  |
|  | *n* | Percent Mated |  | *n* | Percent Mated |  |  |  |
| BC1 | 102 | 81.4 |  | 28 | 92.6 |  | 0.244 | < 0.001  (55.26; 14) |
| BC2 | 84 | 33.3 |  | 33 | 87.9 |  | 0.000 |  |
| BC5 | 32 | 62.5 |  | 3 | 100 |  | 0.536 |  |
| BC6 | 28 | 50.0 |  | 1 | 100 |  | 1.000 |  |
| BC7 | 12 | 66.7 |  | 19 | 89.5 |  | 0.174 |  |
| BC9 | 131^d^ | 83.7 |  | 18 | 88.9 |  | 0.734 |  |
| BC10 | 119 ^d^ | 78.2 |  | 35 | 97.1 |  | 0.003 |  |

^a^Females that were determined to emit the E, Z or H pheromone type (see Table 2).

^b^*p*-values from two-tailed Fisher’s exact tests comparing the percentage of mated females among determined *vs*. undetermined females within each backcross line.

^c^*p*-values, *χ²* values and degrees of freedom (*df*) of two-tailed Fisher's tests for multiple comparisons.

^d^Three females for BC9 and four females for BC10 which were determined with respect to pheromone type but not with respect to mating status were excluded from this count.
