# Supplementary material for: Assortative Mating between European Corn Borer Pheromone Races: Beyond Assortative Meeting
Source: PLoS One. 2007 Jun 20;2(6):e555. doi: 10.1371/journal.pone.0000555 (PMC1891084; doi:10.1371/journal.pone.0000555)
Supplement: Table S2 — Comparison of the mating success of pure-strain (A) females with that of F1 and backcross females that have a father (B) or a mother (C) of the same strain. A and B (respectively C) females are expected to have an identical mating success if Am is Z-chromosome (respectively W-chromosome) linked. The backcrosses are those described in Table 2. (0.06 MB DOC) [file pone.0000555.s002.doc]

| Geo-graphic origin | Test male’s race | A (pure-race) females | | B (F1 or backcross) females  with a father of the same strain as A females | | | | | C (F1 or backcross) females with a mother of the same strain as A females | | | | |
| --- | --- | --- | --- | --- | --- | --- | --- | --- | --- | --- | --- | --- | --- |
|  |  | Strain | %mated (*n*) | Line(s) | %mated (*n*) | Expected mating success^b^ | *p* -value^c^ | Overall *p*-value (*χ²*; *df*) | Line(s) | %mated (*n*) | Expected mating success^d^ | p-value^c^ | Overall *p*-value (*χ²*; *df*)^e^ |
|  |  |  |  |  |  |  |  |  |  |  |  |  |  |
| France | Z | Z | 84.0 (50) | BC1 | 83.8 (130) | A > B | 0.588 | < 0.001 | F1 ^a^, BC1, 5 & 6 | 74.2 (244) | A > C | 0.094 | < 0.001 |
| France | E | Z | 12.0 (50) | BC2, 3 & 4 | 44.7 (152) | A < B | <0.001 | (28.69; 6) | F1 ^a^, BC2, 3 & 4 | 45.5 (202) | A < C | < 0.001 | (18.54; 4) |
| France | Z | E | 36.0 (50) | F1 ^a^, BC5 & 6 | 63.2 (114) | A < B | 0.001 |  |  |  |  |  |  |
|  |  |  |  |  |  |  |  |  |  |  |  |  |  |
| USA | E | Z | 54.0 (50) | BC7 | 82.9 (35) | A < B | 0.005 | < 0.001 | BC11 | 83.3 (55) | A < C | < 0.001 | < 0.001 |
| USA | E | E | 92.3 (52) | F1 ^a^, BC10 | 83.4 (205) | A > B | 0.076 | (29.57; 6) | F1 ^a^, BC7 & 10 | 32.7 (240) | A > C | < 0.001 | (27.63; 4) |
| USA | Z | E | 28.0 (50) | F1 ^a^, BC8 & 9 | 61.9 (231) | A < B | <0.001 |  |  |  |  |  |  |

^a^Obtained from a female Z x male E crosses.

^b^If *Am* is not Z-chromosome linked.

^c^*p*-value of a one-tailed Fisher’s exact test.

^d^If *Am* is not W-chromosome linked.

^e^ *p*-value, *χ²* values and degrees of freedom (*df*) of two-tailed Fisher's tests for multiple comparisons.
